# Metformin Inhibited Proliferation and Metastasis of Colorectal Cancer and presented a Synergistic Effect on 5-FU

**DOI:** 10.1155/2020/9312149

**Published:** 2020-08-10

**Authors:** Jing Sang, Ruixue Tang, Min Yang, Qing Sun

**Affiliations:** ^1^Department of Pathology, Shandong Provincial Qianfoshan Hospital, Cheeloo College of Medicine, Shandong University, Jinan 250014, China; ^2^Department of Pathology, Tai'an Central Hospital, Tai'an 271000, China; ^3^Department of Pathology, The First Affiliated Hospital of Shandong First Medical University, Jinan 250014, China

## Abstract

The purpose of this study was to investigate the effect of metformin or the combination of metformin and 5-FU on the growth and metastasis of colorectal cancer (CRC). For the *in vitro* experiments, HCT 116 and SW1463 cell lines were treated with metformin or the combination of metformin and 5-FU. Cell proliferation and invasion were analyzed by CCK-8, colony formation, and transwell assay, respectively. For the *in vivo* experiments, the CRC xenograft nude mice model was used to observe the effects of metformin or combined with 5-FU on tumor growth and metastasis. Metformin significantly inhibited the proliferation and invasion of HCT116 and SW1463 cells *in vitro*, which showed synergetic effects to 5-FU. In CRC xenograft nude mice, metformin alone and metformin combined with 5-FU treatment significantly inhibited tumor cell proliferation and tumor metastasis. In summary, metformin played an inhibitory role in the proliferation and metastasis of CRC and had a synergistic effect with 5-FU. Metformin may be a potentially effective anti-metastatic drug or an anticancer adjuvant agent for treating CRC.

## 1. Introduction

Colorectal cancer (CRC), one of the most common malignancies, ranks third in terms of cancer incidence and second in terms of mortality, according to the Global Cancer Statistics in 2019 [1]. Nowadays, the mortality of CRC shows decline in the developed countries with the advance of treatment options and organized screening [2]. Nevertheless, the mortality in many developing countries is still rising rapidly [3]. In clinical settings, metastasis is responsible for about 90% cancer-related deaths [4]. All the CRC patients are at risks of metastasis, and approximately 20% of them have already been confirmed with metastasis at first diagnosis in the Netherlands [5]. As one of the main postoperative treatments, chemotherapy is considered to be beneficial to the CRC patients; however, there are indeed drug-resistance and side effects [6, 7]. Therefore, novel drugs and strategies are urgently required to suppress metastasis of CRC and reduce resistance to chemotherapy.

Metformin has been commonly utilized for treating type 2 diabetes mellitus (T2DM) [8]. In the past decades, much attention has been paid to the efficiency of metformin in the treatment of cancers. For instance, *in vitro* and *in vivo* experiments indicated that metformin could inhibit the growth of cancer cells [9–13]. Moreover, it showed antimetastasis effects in a variety of cancer cell lines such as osteosarcoma [14], endometrial cancer [15], and ovarian cancer [16], as well as a few animal models [16–18]. To date, there are still controversies on the effects of metformin on CRC. For example, metformin was reported to be associated with decreased CRC risks and mortality in T2DM patients. Additionally, the association between long-term metformin and CRC gave an adjusted odds ratio (OR) at 0.83 (95% CI 0.68–1.00) [19]. In a retrospective analysis, the estimated 3-year CRC-specific survival rate for the metformin group was significantly higher than that of the nonmetformin group (92.4% vs. 90.8%; log-rank *P* = 0.042) [20]. In contrast, some studies proposed no protective effects after metformin administration on the incidence and survival of CRC [21, 22]. Moreover, most metformin-related studies were conducted on CRC patients combined with diabetes. Rare studies focused on the roles of metformin in CRC patients, especially its antimetastasis effects.

In addition, the effects of metformin combined with different chemotherapeutics have been explored in many cancers. Soichiro Honjgo et al. found that metformin combined with 5-FU significantly sensitized esophageal cancer cells to the cytotoxic effects of 5-FU, including inhibited cell proliferation and induced cell apoptosis [10]. Shu-Ching Hsieh et al. found that the cotreatment of metformin and sorafenib had a synergistic inhibitory effect on hepatocellular carcinoma cell migration and invasion [23]. At present, a few studies have explored the effect of metformin combined with 5-FU on the proliferation of CRC cells [24], but its effect on the invasion and metastasis of CRC cells remains unclear.

In this study, we investigated the effects of metformin or the combination of metformin and 5-FU on the growth and metastasis of CRC *in vitro* and *in vivo*. Our data demonstrated that metformin played an inhibitory role in the proliferation and metastasis of CRC and had a synergistic effect with 5-FU.

## 2. Materials and Methods

### 2.1. Cell Culture

The human CRC lines (i.e., HCT116 and SW1463) were obtained from Genechem (Shanghai, China). The cells were cultured with RPMI1640 medium (Gibco, USA) containing 10% fetal bovine serum (FBS, CLARK, Austria), 1% penicillin, and 1% streptomycin (Hyclone, USA) in a cell incubator at 37°C in 5% CO_2_. Cells in the logarithmic growth phase were used in the experiments. The study protocols were approved by the Ethical Committee of Shandong University.

### 2.2. Cell Proliferation Assay

Cell proliferation was measured using Cell Counting kit-8 (Dojindo, Japan) and colony formation assay. For CCK-8 array, HCT116 and SW1463 cells (3 × 10^3^ cells/well) were seeded onto 96-well plates, followed by culturing for 24 hrs. Then, the mixture was incubated with metformin (0, 1 mM, 5 mM, 10 mM, 20 mM, and 40 mM) or 5-FU (0, 5 *μ*M, 50 *μ*M, 100 *μ*M, 200 *μ*M, and 300 *μ*M) for 24 hrs. Afterwards, CCK-8 reagent (10 *μ*l) was added to each well and incubated for 2 hrs. The absorbance values were measured at a wavelength of 450 nm by using an auto-microplate reader, in order to calculate the cell viability. For the colony formation array, the trypsinized cells (5 × 10^2^ cells/well) were seeded onto 6-well plates. After culturing for 24 hrs, the medium should be replaced with fresh medium containing metformin (5 mM) with or without 5-FU (5 *μ*M), respectively. Upon the presence of clones, cells were fixed with methanol for 15 min and stained with 1% crystal violet. The total number of colonies containing >50 cells was counted. Metformin and 5-FU were purchased from Sigma-Aldrich (St. Louis, USA) and dissolved in phosphate-buffered saline (PBS) (Shanghai, China).

### 2.3. Cell Invasion Assay

Cell invasion was measured by Transwell assay using 24-well chambers (BD, USA) with 8 mm pore polycarbonate membrane insert coated with 100 *μ*l of Matrigel (BD Biosciences). At first, cells (1 × 10^5^) resuspended in FBS-free medium (100 *μ*l) were added into the upper chamber. Then, the medium (600 *μ*l) fixed with 10% FBS was added into the lower chambers. Upon treating with metformin (5 mM) and/or 5-FU (5 *μ*M) for 24 hrs, cells in the upper chambers were gently wiped with cotton swabs. The membrane invaded by cells were fixed in methanol and then was stained with 1% crystal violet. The cell invasion was observed under an inverted microscope by randomly selecting five visual fields for each chamber. Finally, the average number of cells was calculated.

### 2.4. CRC Xenograft Nude Mice Model

Male BALB/c nude mice (5-week old, *n* = 25), purchased from Huafukang Biotech (Beijing, China), were housed in a sterile environment. All animal experiments were approved by the Animal Ethics Committee of Shandong University. CRC model was established by orthotopic implantation. To be specific, HCT116 cells (2 × 10^7^ cells/ml) resuspended in RPMI 1640 (200 *μ*l) were injected subcutaneously into the left flank of nude mice. A viable solid tumor was established about 5 weeks after injection.

The subcutaneous tumor tissues were dissected into 1 mm^3^ pieces for the subsequent transplantation. For implantation, mice were anesthetized by intraperitoneal injection of 10% chloral hydrate, and then, the abdomen was sterilized with 75% alcohol. An incision (0.5 cm) was made in the left lower abdomen to pull out the cecum. The serosa at the site of implantation was removed. The tumor tissues (1 mm^3^) were sutured on the wall of the cecum by 8-0 surgical suture. Subsequently, the cecum was refolded to the abdominal cavity, followed by skin suturing.

One week after transplantation, the mice were randomly divided into four groups: (a) control group (*n* = 5), received intraperitoneal injection of 0.9% sodium chloride; (b) metformin group (*n* = 5), received oral lavage of metformin (250 mg/kg) per day; (c) 5-FU group (*n* = 5), received intraperitoneal injection of 5-FU (25 mg/kg) once a week; (d) cotreatment group (*n* = 5), received oral lavage of metformin (250 mg/kg) per day combined with 5-FU (25 mg/kg) via intraperitoneal injection once a week. Four weeks after treatment, all mice were sacrificed after cervical dislocation followed by measuring the tumor growth and observation of metastasis. The tumor volume (mm^3^) was calculated according to the previous description with the formula: 0.5 × length × width^2^ [25]. All orthotopic and metastatic tumors were fixed in the neutral formalin for pathological examination.

### 2.5. Immunohistochemistry

The paraffin-embedded tumor tissues (4 *μ*m) were subject to deparaffinization, dehydration, and antigen retrieval and block, respectively. Then, the slices were incubated with Ki-67 antibody (MXB Biotech, China) at 4°C overnight, together with HRP-conjugated secondary antibodies at 37°C for 45 min. Daminoben-zidine tetrahydrochloride (DAB) (MXB Biotech, China) was used for color development, and hematoxylin was used for counterstained. Finally, the staining results were observed under a light microscope. Only unequivocal nuclear staining was counted regardless of its intensity. The fraction of cells with positive staining for anti-Ki-67 was quantitated [26].

### 2.6. Statistical Analysis

SPSS 16.0 software (IBM, USA) was used for the data analysis. Quantitative data were presented as the mean ± standard deviation. One-way ANOVA and Student's *t*-test were utilized for the comparison of intergroup differences. Enumeration data were analyzed by Pearson's chi-square test or Fisher's Exact Test. *P* < 0.05 was considered to be statistically significant.

## 3. Results

### 3.1. Metformin Inhibited CRC Cell Proliferation and Enhanced the Antiproliferation Effect of 5-FU *In Vitro*

**Figure 1:**
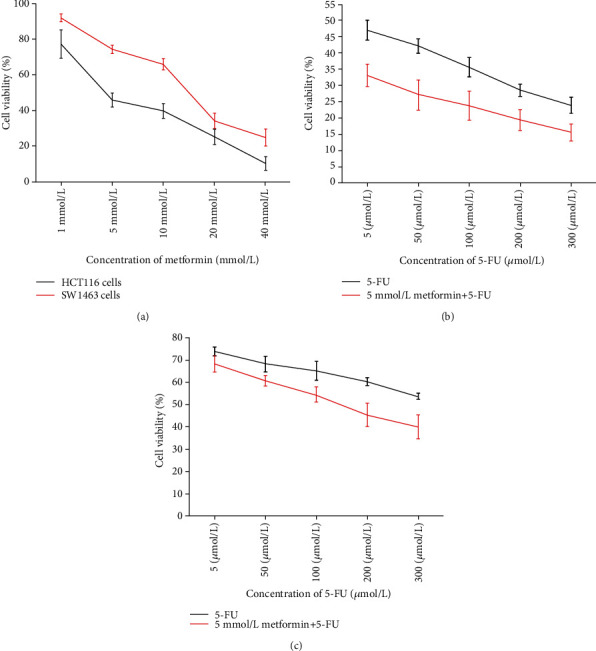
Metformin and the combination of metformin and 5-FU inhibited the viability of HCT116 and SW1463. Metformin inhibited the proliferation of HCT116 and SW1463 cells in a dose-dependent manner (a). Metformin (5 mM) combined with 5-FU could significantly inhibit the proliferation of the HCT116 (b) and SW1463 (c) cells compared to the 5-FU (*P* < 0.05).

**Figure 2:**
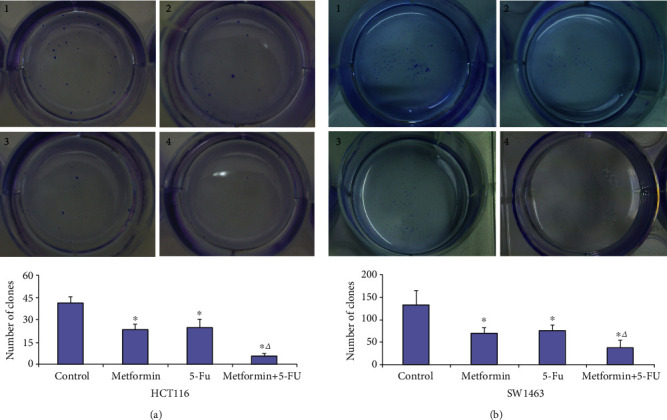
Metformin and the combination of metformin and 5-FU inhibited the colony formation in HCT116 (a) and SW1463 cells (b). 1: control; 2: 5 mM metformin; 3: 5 *μ*M 5-FU; 4: 5 mM metformin+5 *μ*M 5-FU. ∗*P* < 0.05 compared with control group; ^△^*P* < 0.05 compared with 5-FU group.

To investigate the effects of metformin and/or 5-FU on CRC cell proliferation, CCK-8 assay and colony formation assay were performed. In metformin group, metformin could inhibit the proliferation of HCT116 and SW1463 cells in a dose-dependent manner (Figure 1(a)). Compared with 5-FU group, significant inhibition was observed in the cell proliferation of HCT116 cells (*P* = 0.001) and SW1463 cells (*P* = 0.004) after treating with the combination of 5 mM metformin and 5 *μ*M 5-FU (Figures 1(b) and 1(c)). The 50% inhibitory concentration (IC_50_) of metformin in HCT116 and SW1463 cells was 4.6 mM and 13.6 mM, respectively. The selected concentrations for metformin (5 mM) and 5-FU (5 *μ*M) were approximately IC_50_ of HCT 116 cells, while the concentrations for metformin (5 mM) and 5-FU (5 *μ*M) were approximately IC_25_ of SW1463 cells. The results of colony formation assay revealed that 5 mM metformin significantly inhibited the formation of colonies in HCT116 (*P* = 0.004) and SW1463 (*P* = 0.032) cells. Compared with 5-FU group, 5 mM metformin combined with 5 *μ*M 5-FU significantly inhibited the formation of colonies in HCT116 (*P* = 0.005) and SW1463 (*P* = 0.037) cells (Figure 2).

### 3.2. Metformin Inhibited CRC Cell Invasion and Synergized with 5-FU *In Vitro*

**Figure 3:**
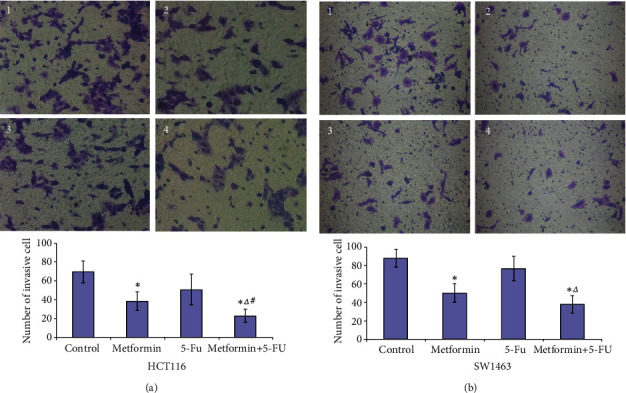
Metformin and the combination of metformin and 5-FU inhibited the invasion of HCT116 (a) and SW1463 cells (b) *in vitro*. (a) The images were observed under a magnification of 200x. 1: control; 2: 5 mM metformin; 3: 5 *μ*M 5-FU; 4: 5 mM metformin+5 *μ*M 5-FU. ∗*P* < 0.05 compared with control group; ^△^*P* < 0.05 compared with 5-FU group. ^#^*P* < 0.05 compared with metformin group.

Transwell assay demonstrated that the number of invasive cells in metformin group was significantly lower than that of control group (HCT116 cells, *P* = 0.002; SW1463 cells, *P* < 0.001). Additionally, in cotreatment group, the decline of the number of invasive cells was much more noteworthy compared with that of the 5-FU group (HCT116 cells, *P* = 0.008; SW1463 cells, *P* = 0.001). Compared with metformin group, the combination of metformin (5 mM) and 5-FU (5 *μ*M) could significantly decrease the number of invasion cells in HCT116 cells (*P* = 0.019). However, there was no statistical differences in the number of invasive cells in cotreatment group compared with that of the metformin group in SW1463 cells (*P* > 0.05). In addition, there was no statistical differences in the number of invasive cells in 5-FU group compared with that of the control group (*P* > 0.05, Figure 3).

### 3.3. Metformin and the Combination of Metformin and 5-FU Inhibited Proliferation and Metastasis of CRC *In Vivo*

**Figure 4:**
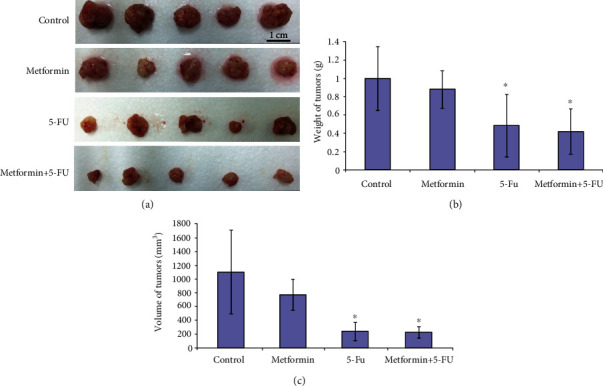
Effects of Metformin and the combination of metformin and 5-FU on the CRC tumor weight and volume *in vivo*. (a–c) Tumors, tumor weight, and tumor volume in each group (*n* = 5). ∗*P* < 0.05 compared with control group.

All the mice showed the presence of tumors 5 weeks postorthotopic implantation. Compared with the control group, the orthotopic tumor volume showed significant decline in the 5-FU group (237.6 ± 131.3 mm^3^ vs. 1097.9 ± 603.6 mm^3^, *P* = 0.032) and the co-treatment group (224.5 ± 79.3 mm^3^ vs. 1097.9 ± 603.6 mm^3^, *P* = 0.031). No significant differences were found in the orthotopic tumor volume between the metformin group and control group (768.3 ± 224.0 mm^3^ vs. 1097.9 ± 603.6 mm^3^, *P* = 0.285). Besides, no statistical differences were noticed in the orthotopic tumor volume between cotreatment group and the 5-FU group (224.5 ± 79.3 mm^3^ vs. 237.6 ± 131.3 mm^3^, *P* = 0.853). Similarly, compared with the control group, the orthotopic tumor weight showed significant decline in the 5-FU group (0.48 ± 0.34 g vs. 1.00 ± 0.35 g, *P* = 0.046) and the cotreatment group (0.42 ± 0.24 g vs. 1.00 ± 0.35 g, *P* = 0.016). No significant differences were found in the orthotopic tumor weight between the metformin group and control group (0.88 ± 0.20 g vs. 1.00 ± 0.35 g, *P* = 0.537). Besides, no statistical differences were noticed in the orthotopic tumor weight between cotreatment group and the 5-FU group (0.42 ± 0.24 g vs. 0.48 ± 0.34 g, *P* = 0.743, Figure 4).

**Figure 5:**
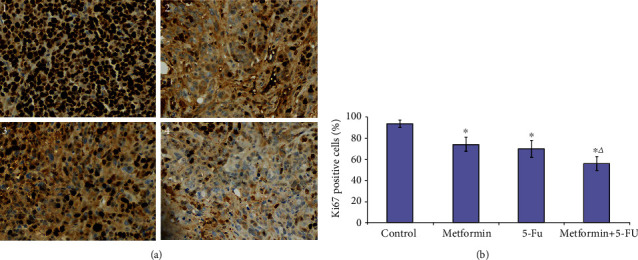
Metformin and the combination of metformin and 5-FU inhibited the proliferation of CRC *in vivo*. (a). Immunohistochemical staining of Ki67 in each group. The images were observed under a magnification of 200x. (b). Quantification of Ki67-positive cells in each group. 1: control; 2: metformin; 3: 5-FU; 4: metformin+5-FU. ∗*P* < 0.05 compared with control group); ^△^*P* < 0.05 compared with 5-FU group.

In our study, the proliferation of cancer cells in the orthotopic implantation tumor was also evaluated by immunohistochemical staining of Ki-67. The proportion of Ki-67 positive cells in the metformin group and the 5-FU group was significantly lower than that of the control group (metformin group vs. control group, *P* < 0.001; 5-FU group vs. control group, *P* < 0.001). In addition, the proportion of Ki-67 positive cells in the cotreatment group was significantly lower compared with the 5-FU group (*P* = 0.016, Figure 5).

**Figure 6:**
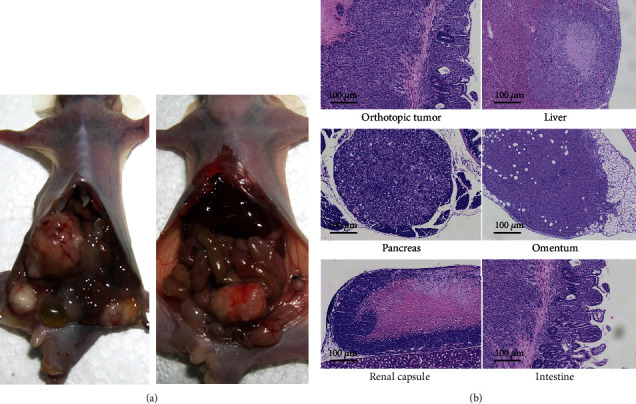
Metformin and the combination of metformin and 5-FU inhibited the metastasis of CRC *in vivo*. (a). Representative macroscopic images of CRC with or without metastasis. (b). Representative photomicrographs of metastatic carcinoma. The images were observed under a magnification of 100x.

Visual and microscopic examinations revealed that there were multiple distant metastases in all control mice (5/5) and 80% of the mice in 5-FU group (4/5). Metastatic sites/organs included pancreas, liver, intestine, omentum, and renal capsule. Mice in the metformin group (1/5, 20%) and cotreatment group (1/5, 20%) only showed liver metastasis. Compared with control group, the distant metastatic rate showed significant decline in metformin group (*P* = 0.048) and cotreatment group (*P* = 0.048), respectively. No significant differences were noticed in the distant metastasis rate between the cotreatment group and the 5-FU group (*P* > 0.05, Table 1, Figure 6).

## 4. Discussion

It is estimated that by 2030, there will be more than 2.2 million CRC cases and 1.1 million cancer deaths worldwide [27]. For stage I and II CRC patients, the 5-year relative survival rate were 91% and 82%, respectively. However, the 5-year survival rate for stage IV patients decreased to 12% in the US [28]. Nowadays, surgery, chemotherapy, and radiation therapy are the main treatments for the CRC patients, and for a long time, many researchers have been committed to the development and research of new drugs. Recently, metformin, a common hypoglycemic drug, has attracted more attention for its antitumor effects. There are disputes on the effects of metformin on CRC. Besides, the effects of metformin combined with chemotherapeutics on CRC are not clear. Therefore, in this study, *in vitro* and *in vivo* experiments were conducted to comprehensively investigate the effects of metformin and/or 5-FU on the growth and metastasis of CRC. Our data demonstrated that metformin played inhibitory roles in the proliferation and metastasis of CRC. Besides, it presented synergistic effects on the antiproliferation activity and anti-invasion captivity of 5-FU.

Currently, 5-FU has been widely used as a chemotherapy agent for treating CRC; however, its clinical efficiency is hampered to some extent due to drug resistance of cancer cells [29]. In this study, we investigated the anticancer effects of the combination of 5-FU and metformin, with 5-FU serving as the positive control. As previously described, metformin has been shown to inhibit the proliferation and enhance chemosensitivity in a variety of cancer cell lines, such as esophageal cancer [10], breast cancer [30], intrahepatic cholangiocarcinoma [31], and ovarian cancer [17]. Additionally, it was reported to show inhibitory effects on the invasion and migration of cancer cells [18, 23, 32]. Our data revealed that metformin significantly restricted the proliferation and invasion of HCT116 and SW1463 cells. Besides, metformin combined with 5-FU had synergistic effects, which was featured by enhanced reduction to the proliferation and invasion of these cells. Previous studies on CRC showed that metformin could enhance the antiproliferative effects of 5-FU on SW620 cells [24]. Also, it could promote the antiproliferative effects of silibinin on COLO 205 cells [33]. This study results were consistent with these.

To the best of our knowledge, this is the first study to explore the effects of metformin on the growth and metastasis of CRC by orthotopic implantation in nude mice model. Compared with subcutaneous tumor implantation, such animal model contributed to the investigation on the biology of colon cancer metastatic capability and the development of new drugs active against metastatic cancer [34]. In our study, all the mice developed tumors five weeks postorthotopic tumor implantation. After 4 weeks of metformin treatment, the tumor cell proliferation (indicated by Ki-67 staining) significantly decreased. More importantly, our study demonstrated that metformin significantly inhibited colon cancer metastasis. 5-FU had significant inhibitory effects on the cancer cell proliferation other than invasion *in vitro*. However, when combined with metformin, the cancer cell invasion was significantly inhibited compared with the control group and the 5-FU group (*P* < 0.05). Under *in vivo* conditions, 5-FU showed no inhibition on the colon cancer metastasis. Nevertheless, compared with the control group, it showed inhibitory effects when combing with metformin. No statistical differences were noticed in the metastasis between the 5-FU group and the group subject to the combination of 5-FU and metformin. The selected dosage of metformin in this study was calculated according to the formula, i.e., human equivalent dose (mg/kg) = animal dose (mg/kg) × animal Km/human Km (Km values are based on body surface area) [35]. A metformin dose of 250 mg/kg in mouse was equivalent to 1216 mg in a person of 60 kg, which was far less than the maximal safe dose of 2,550 mg/d. In a previous study, intraperitoneal injection of metformin (250 mg/kg) every other day for 4 weeks inhibited hepatic, intestinal and lung metastasis in ovarian cancer [16]. Additionally, in orthotopic mouse with hepatocellular carcinoma, oral administration of metformin (200 mg/kg, qd) for 37 days combined with sorafenib (30 mg/kg) significantly decreased the postoperative metastases [36]. In general, these different dose and administration methods of metformin resulted in similar effects on different tumors. In future, further studies are required to determine the appropriate and safe dose of metformin for various malignancies.

Our study still has some limitations. On the one hand, due to technical limitations, no lymph node metastasis was observed *in vivo*. On the other hand, there are no studies focusing on the elucidation of the precise mechanism underlying antimetastasis effect of metformin in CRC. In future, we will focus on these issues.

In summary, metformin showed inhibitory effects on the proliferation and metastasis of CRC under *in vitro* and *in vivo* conditions. Besides, it showed synergistic effects on the anticancer activity of 5-FU. The combination of metformin and chemotherapeutic agents may provide new regimens to attenuate or even eliminate the drug resistance and side effects.

## Figures and Tables

**Table 1:** The distant metastatic rate of four groups.

Group	Distant metastatic rate	*P* value
Control	5/5(100%)	
Metformin	1/5(20%)	0.048^∗^
5-FU	4/5(80%)	1.000
Metformin+5-FU	1/5(20%)	0.048^∗^

^∗^Compared with control group.

## Data Availability

All the data were available upon appropriate request.
